# Long-term Results of Topical Insulin Treatment for Persistent Corneal Epithelial Defects

**DOI:** 10.18502/jovr.v19i4.13977

**Published:** 2024-12-31

**Authors:** Júlio Almeida, Tomás R. Costa, Maria Vivas, Catarina Monteiro, Fernando T. Vaz, Diana Silva, Cristina Vendrell, Isabel Prieto

**Affiliations:** ^1^Ophthalmology Department, Prof. Doutor Fernando Fonseca Hospital, EPE, Amadora, Portugal; ^3^https://orcid.org/0000-0002-4651-8812

**Keywords:** Cornea, Corneal ulcer, Herpetic Keratitis, Insulin, Keratitis

## Abstract

**Purpose:**

To evaluate the effects of topical insulin in patients with persistent corneal epithelial defects that are refractory to the standard treatment.

**Methods:**

A retrospective, hospital-based, clinical study was performed on 17 eyes of 16 patients with different types of refractory persistent epithelial defects who were treated with topical insulin. The treatment was continued until the defect either was resolved or persisted after three months. Patients' demographic information, etiology, comorbidities, and clinical data were reviewed. The rate of epithelial healing was considered as the primary outcome measure.

**Results:**

Neurotrophic keratitis was the most common cause of persistent epithelial defects (58.8%), and within this category, herpetic eye disease was the main comorbidity (44.4%). The mean follow-up time was 17.91 months. Eleven out of fifteen eyes (77.3%) had complete improvement and only one patient did not respond to the treatment. The mean time of reepithelization for the eyes with full recovery was 31.27 days (ranging from 6 to 61 days). The best-corrected visual acuity improved significantly after treatment (*P* < 0.005), and there were no reports of complications or side effects during the study period.

**Conclusion:**

Our results suggest that topical insulin, due to its good safety profile, availability, and affordability, could be a good therapeutic alternative for persistent epithelial defects.

##  INTRODUCTION

The cornea has two fundamental roles: acting as the main barrier to external aggressors and being one of the major refractive interfaces of the eye.^[1,2]^ Disturbance in this layer's epithelium can lead to devastating consequences to the eye's overall health, from vision loss to infection and ulceration.^[2,3]^ Corneal erosions and epithelial defects are among the most common pathologies in the ophthalmology emergency room.

The corneal epithelium is highly rich in several growth factors that are essential in the healing process of injuries. Any disruption to this process can transform an acute defect into a persistent epithelial defect (PED),^[2]^ which is defined as any superficial corneal defect that does not heal after more than 10 to 14 days, even with conventional supportive treatment.^[2,4]^

PEDs have multiple etiologies, from infection to trauma, medications, limbal cell deficiency, and poor epithelial adhesion. One of the most common causes is neurotrophic keratopathy, which is a degenerative disorder characterized by impaired corneal innervation and is often triggered by a previous corneal herpetic infection.^[5]^

The current management of PEDs is challenging and usually requires multiple actions depending on the cause. Supportive care is the mainstay of treatment and involves intensive preservative-free lubrication, bandage soft contact lenses, punctal plugs, prophylactic topical antibiotics, and treatment of the underlying condition. Further treatment options include hemoderivatives such as autologous serum drops and more invasive surgical approaches like amniotic membrane transplant.^[2]^ Despite their good results, autologous serum drops have some drawbacks, including the price, the complexity of preparing previous blood samples, and the need for qualified pharmacists.^[6]^

More recent treatments are especially focused on corneal regeneration. There is an increasing use of growth factor analogues or enhancers such as the recombinant human epidermal growth factor (EGF) and recombinant human nerve growth factor (cenegermin).^[2,7]^ Despite their potential therapeutic effects, these agents have some limitations, including the high cost of cenegermin and the limited access to both products.^[8,9]^ Insulin-like growth factor (IGF) is another crucial growth factor in the proliferation and differentiation of corneal epithelium cells. It exerts its effects through insulin and IGF receptors.^[10]^

Several studies have reported the potential effect of topical insulin in the treatment of PED.^[7,10,11,12,13,14,15,16]^ The treatment of PEDs is generally difficult and expensive, making them a good candidate for novel drugs that enhance and promote corneal regeneration. This study aims to help establish the role of topical insulin in treating PEDs that are refractory to standard treatment.

##  METHODS

This retrospective, single-center study was performed at Prof. Doutor Fernando Fonseca Hospital, Amadora, Portugal. It involved patients with PED who had been treated with topical eye insulin drops from April 2021 to April 2023.

The study adhered to the ethical guidelines of the Declaration of Helsinki, and it was approved by the Ethics Commission for Health at the Prof. Doutor Fernando Fonseca Hospital (approval code 70/2023).

The study included adult patients with different types of PED in one or both eyes. We defined a PED as a corneal epithelial defect that was refractory to standard support treatment for more than 14 days.^[4]^ Patients with active ocular infection and anticipated need for surgical treatment (amniotic membrane transplant or tarsorrhaphy) were excluded from the study. No control group was included in this study. All patients were informed about the risks, benefits, and therapeutic alternatives. Also, a written informed consent was obtained for the off-label use of topical insulin.

**Table 1:** Summary of patients' characteristics.

***Patient (eyes)***	***Age (yrs)***	***Sex***	***PED etiology***	***Prior topical treatments***	***Pre-treatment BCVA*** ***(logMAR)***	***Post-treatment BCVA (logM*** **AR** ***)***	***Time to complete reepithelization (days)***	***Recurrence***
*1*	35	Male	Post-herpetic neurotrophic keratitis	Corticosteroids Lubricants	0.4	0.1	20 (partial)	No ${\;}^{†}$
*2*	95	Female	Post-herpetic neurotrophic keratitis	Antibiotics Lubricants	–	–	83 (partial)	No
*3*	56	Female	Post-infectious neurotrophic keratitis	Antibiotics Lubricants	–	–	55 (partial)	Yes
*4*	75	Female	Primary severe dry eye disease	Cyclosporine 0.1% Lubricants	0.7	0.6	56	No
5*	46	Male	Exposure keratopathy	Antibiotics Corticosteroids Lubricants	0.4	0.2	61	No
6*	46	Male	Exposure keratopathy	Antibiotics Lubricants	0.7	0.4	6	No
*7*	82	Male	Diabetes Mellitus-associated neurotrophic keratitis	Antibiotics Corticosteroids Lubricants	1.5	1.3	27	No
*8*	29	Male	Mooren's ulcer	Cyclosporine 0.1% Antibiotics Corticosteroids Lubricants Bandage contact lens	0.4	0.3	7	Yes
*9*	89	Female	Secondary severe dry eye disease	Antibiotics Corticosteroids Lubricants Bandage contact lens	1	0.8	27	No
*10*	85	Female	Limbal stem cell insufficiency	Corticosteroids Lubricants	0.7	0.3	55	No
*11*	84	Female	Diabetes Mellitus-associated neurotrophic keratitis	Antibiotics Corticosteroids Lubricants Bandage contact lens	0.7	0.3	7	No
*12*	56	Female	Post-surgical neurotrophic keratitis	Antibiotics Corticosteroids Lubricants	0.9	1	18	No
*13*	73	Female	Post-herpetic neurotrophic keratitis	Corticosteroids Lubricants	1.3	0.1	27	No
*14*	60	Male	Exposure keratopathy	Antibiotics Corticosteroids Lubricants	1.3	1.3	53	No
*15*	87	Male	Rosacea associated keratitis	Cyclosporine 0.1% Corticosteroids Lubricants	–	–	–	–
*16*	61	Female	Post-herpetic neurotrophic keratitis	Corticosteroids Lubricants Bandage contact lens	–	–	(Abandoned)	–
*17*	59	Female	Post-infectious neurotrophic keratitis	Antibiotics Corticosteroids Lubricants	–	–	(Abandoned)	–
*Same patient, different eyes; ${\;}^{†}$ Reactivation of the herpetic keratitis; PED, persistent epithelial defect; BCVA, best-corrected visual acuity								

**Table 2:** Reepithelization results in patients treated with topical insulin.


**Epithelization results**	**Topical insulin eye drops (** ***n*** ** = 15)**
Total reepithelization [textitN (%)]	11 (73.3%)
Time till reepithelization (days)	
Mean ± SD	31.7 ± 21.4
Range	6–61
Recurrence [textitN (%)]	2 (13.3%)
Pre-treatment BVCA [logMAR (mean ± SD)]	0.83 ± 0.37
Pre-treatment BVCA [logMAR (mean ± SD)]	0.56 ± 0.44
Follow-up time (months)	
Mean ± SD	17.91 ± 7.63
Range	2.66 – 23.82
n, number; SD, standard deviation	

**Figure 1:**
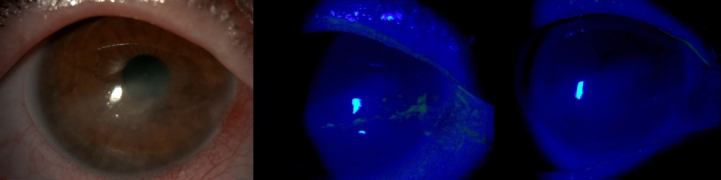
Slit-lamp follow-up images of patient no 6. A 46-year-old male with exposure keratopathy after long-term hospitalization due to COVID-19 pneumonia. (a & b) Irregular epithelial defects before treatment with topical insulin. (c) Six days of topical insulin leading to complete recovery of corneal epithelium.

**Figure 2:**
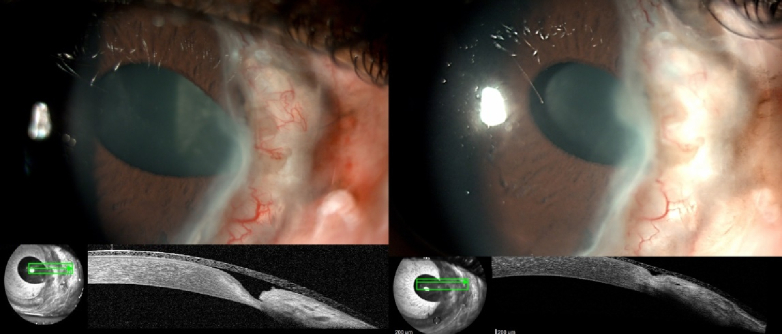
Slit-lamp and anterior segment optical coherence tomography images of patient no 8 at follow-up. A 29-year-old male with a history of Mooren's ulcer, under both topical and oral corticosteroids and cyclosporin. (a & b) Deep epithelial defect even after bandage contact lens placement. (c & d) Seven days of topical insulin leading to complete recovery of corneal stroma and epithelium.

**Figure 3:**
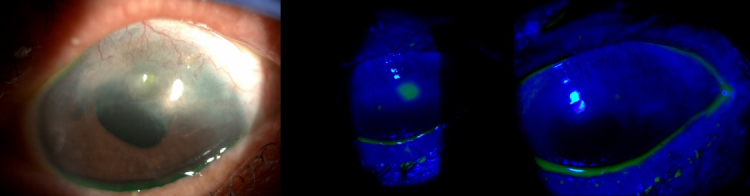
Slit-lamp follow-up images of patient no 11. An 84-year-old female patient with diabetes and chronic glaucoma who developed neurotrophic keratitis. (a & b) Superior epithelial defect associated with a possible vascular pannus. (c) Seven days of topical insulin leading to complete recovery of corneal epithelium.

Topical insulin eye drops were prescribed to all patients four times daily. Drops were prepared using a sterile technique in the hospital's Pharmacy Department by injecting short-acting insulin into a bottle of artificial tears with propylene glycol base in a concentration of 1 unit per milliliter (IU/mL). The final preparation had to be refrigerated and had a shelf life of one month. Each patient was given detailed written instructions.

All patients received multiple clinical visits to assess the evolution of the treatment. The initial appointment before starting treatment consisted of collecting demographic data, reviewing the patient's medical history, performing a slit-lamp examination, and documenting the PED characteristics. The subsequent examinations were conducted at weeks 1, 2, and 4, followed by monthly visits until the defect healed. Insulin treatment was stopped if there was no response after three months.

The rate and time of epithelial healing were considered the primary outcomes. Time to reepithelization was reported in days so as to ensure standardization with other published data. Secondary outcomes included the number of patients with complete resolution of the PED and improvement in visual acuity.Statistical analysis was conducted using SPSS. The descriptive data were presented as mean, median, standard deviation, and range.

##  RESULTS

From April 2021 to April 2023, a total of 17 eyes from 16 patients (10 females and 6 males) were treated with topical insulin eye drops. The mean age of this population was 65.76 ± 19.88 years. The mean follow-up time was 17.91 ± 7.63 months after starting treatment. Two patients abandoned the study prematurely: the first due to poor adherence and the other due to missing follow-up appointments.

In terms of etiology, neurotrophic keratitis was the most common cause of PED (10 eyes, 58.8%). Within this category, herpetic eye disease was the main comorbidity (4 eyes, 23.5%), followed by diabetes mellitus (2 eyes, 11.8%), postinfectious keratitis (2 eyes, 11.8%), postoperative keratitis (1 eye, 5.9%) and primary severe dry eye syndrome (1 eye, 5.9%). Other important causes were chronic ocular surface inflammation (3 eyes, 17.6%), secondary severe dry eye syndrome (1 eye, 5.9%), Mooren's ulcer (1 eye, 5.9%), rosacea (1 eye, 5.9%), exposure keratopathy (3 eyes, 17.6%), and limbal stem cell deficiency (1 eye, 5.9%).

Of the 15 eyes that remained in the study, 11 (73.3%) had complete resolution of the PED, 3 (20%) had significant but incomplete improvement, and 1 (6.6%) with the shortest follow-up time (2.66 months) did not respond to the treatment. The mean time of reepithelization was 31.27 ± 21.4 days (range 6–61 days) for eyes with full recovery and 52.67 ± 31.56 days for other eyes who did not fully recover. The mean pretreatment best-corrected visual acuity (BCVA) was 0.83 ± 0.37 logMAR, and the mean posttreatment BCVA was 0.56 ± 0.44 logMAR. There was a statistically significant improvement in BCVA (*P* < 0.005) after treatment with topical insulin.

Two patients (13.3%) experienced a recurrence of the PED. One of them developed a new infectious corneal ulcer in the same eye one month after discontinuing topical insulin. The patient did not recover and died a few months later due to COVID-19-associated pneumonia. The other patient experienced a reactivation of the underlying condition (Mooren's ulcer) a year after insulin treatment due to poor adherence systemic treatment. Although not a recurrence, the herpetic eye disease was reactivated in one patient during the insulin treatment, but it resolved after increasing the dose of antivirals.

Treatment with topical insulin was well tolerated and there were no reports of complications or side effects during the time of the study.

##  DISCUSSION

If not properly diagnosed and treated, PEDs can lead to potentially severe complications such as perforation, corneal scarring, and substantial vision loss. The variable success rates of available medical treatments and the more invasive nature of surgical rescue procedures highlight a gap in the management of PEDs that needs to be addressed.

PEDs can now be treated by different methods, including lubrication, bandage contact lens, autologous serum eye drops, and surgery (tarsorrhaphy and amniotic membrane transplant). Topical insulin has been used as a promoter of corneal reepithelization since 1945, when Aynsley et al published the first case series of five patients with refractory epithelial defects.^[17]^ After that, it was only in 2017 when Wang et al published another case series reporting completed reepithelization for six patients with refractory neurotrophic defects.^[18]^

A recent review identified nine studies conducted on over 180 patients between 2013 and 2022. However, only two of these were prospective trials and among them, one study was a randomized controlled trial. According to the review, all cases achieved complete resolution of the PED within 2.5 to 60.9 days.^[19]^

The recent studies by Diaz-Valle et al and Soares et al represent the largest series to date; each focused on 21 patients and observed similar outcomes, with complete reepithelization within 34.8 ± 29.9 and 29 ± 11 days, respectively.^[7,15]^

Regarding autologous serum-based eye drops, the most recent review by the American Academy of Ophthalmology found four articles on PEDs. All of these studies confirmed a significant improvement in the epithelial defects, and three of them reported over 90% reduction in defect size.^[20]^ Diaz-Valle et al (2022) were the first to compare the effects of topical insulin and autologous serum-based drops. They found better epithelization results in the topical insulin group (84% of patients within 32.6 ± 28.3 days) compared to the group receiving autologous serum-based drops (48% of patients within 82.6 ± 82.4 days).^[21]^

On the other hand, two recent randomized trials evaluated and compared the efficacy of cenegermin and vehicle treatment in patients with neurotrophic PEDs. Bonini et al and Pflugfelder et al, respectively, reported complete corneal healing rates of 56.5–58% after four weeks and 69.6–74% after eight weeks of treatment. Both studies showed good tolerability.^[22,23]^ To date, there is no comparative study between cenegermin and topical insulin eye drops.

In our retrospective, non-comparative study, topical insulin eye drops were a viable option for reepithelization of different types of PED. The excellent overall profile, wide availability, low production cost, and absence of local or systemic side effects make topical insulin a great candidate for different applications [Figures 1–3].

Our case series supports clinical long-term results of topical insulin in healing corneal defects of various etiologies. Although the PED resolved in only about 73.3% patients, it showed a significant reduction in nearly all cases within a relatively short period of time (mean of 31.7 days). Only one patient did not respond to the treatment, mainly due to the short follow-up period and the complex pathophysiology of the underlying PED.

To this date, the present study is the only known case series with the longest follow-up period of almost one and a half years (17.91 months). Furthermore, we observed no side effects or complications associated with the treatment, which is in line with the published literature.^[7,12,14,15,18,19,21]^

There are some important limitations in this study. First, this is a retrospective case series with a relatively small number of patients, and there was no control group, which would ensure a fair comparison. Additionally, our sample comprised patients with PEDs of different etiologies. Although we used the same treatment protocol (topical insulin four times a day) in all patients, the adjuvant therapies tailored to the underlying PED etiology introduced some variability in the treatment protocols within our sample.

In summary, topical insulin eye drops are a relatively new, safe, affordable, and easily available alternative to treat PEDs. Hence, they may become integral in the treatment algorithm of this group of challenging diseases that urgently need newer treatment strategies. The results of our study are fairly consistent with existing research; however, larger comparative and prospective studies should be developed to understand the real value of topical insulin.

##  Financial Support and Sponsorship

None.

##  Conflicts of Interest

None.
